# Supplementary material for: A single amino acid substitution in the capsid protein of Zika virus contributes to a neurovirulent phenotype
Source: Nat Commun. 2023 Oct 26;14:6832. doi: 10.1038/s41467-023-42676-7 (PMC10603150; doi:10.1038/s41467-023-42676-7)
Supplement: Supplementary file 1 — Supplementary Information [file 41467_2023_42676_MOESM1_ESM.pdf]

Supplementary Information

A single amino acid substitution in the capsid protein of Zika virus contributes to a neurovirulent phenotype

Guang-Yuan Song[1,2,#], Xing-Yao Huang[2,#], Meng-Jiao He[2,#], Hang-Yu Zhou[3,#], Rui-Ting Li[2], Ying Tian[1,2], Yan Wang[4], Meng-Li Cheng[2], Xiang Chen[2], Rong-Rong Zhang[2], Chao Zhou[2], Jia Zhou[2], Xian-Yang Fang[5], Xiao-Feng Li[2,*], Cheng-Feng Qin[1,2,*]

[1]School of Basic Medical Sciences, Anhui Medical University, Hefei, Anhui 230032, China.

[2]Department of Virology, State Key Laboratory of Pathogen and Biosecurity, Beijing Institute of Microbiology and Epidemiology, Academy of Military Medical Sciences, Beijing 100071, China.

[3]Suzhou Institute of System Medicine, Chinese Academy of Medical Sciences & Peking Union Medical College, Suzhou, Jiangsu 215123, China.

[4]Beijing Advanced Innovation Center for Structural Biology, School of Life Sciences, Tsinghua University, Beijing 100084, China.

[5]Key Laboratory of RNA Biology, Institute of Biophysics, Chinese Academy of Sciences, Beijing 100101, China

[#]These authors contributed equally.

*Correspondence: Xiao-Feng Li (xiaofeng_li_bj@163.com); Cheng-Feng Qin (qincf@bmi.ac.cn).

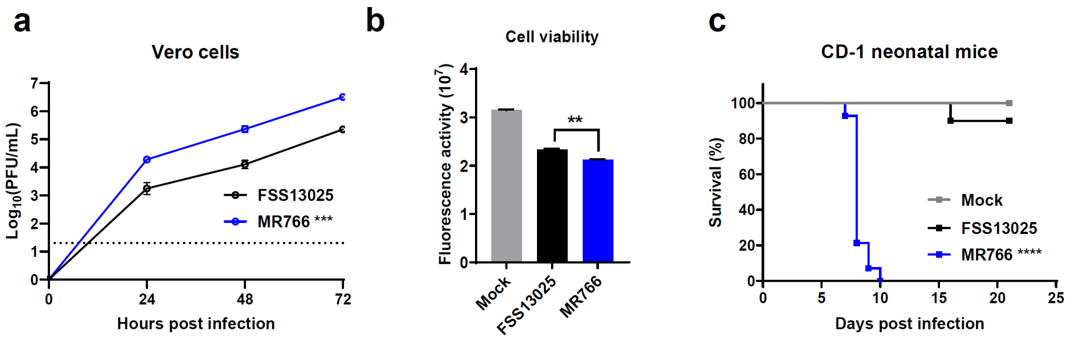

Fig. S1. **a** Growth curves of FSS13025 and MR766 in Vero cells. The cells were infected with FSS13025 or MR766 at an MOI of 0.1. L.O.D., limit of detection. Two-way ANOVA was performed for statistical analysis. ***, $P < 0.001$. **b** Vero cell viability were detected after inoculate with FSS13025 or MR766 at an MOI of 0.01, with DMEM media for Mock in the meantime. The viability was detected at 48 h post infection. Data of growth curves and the cell viability represent the means and SD from one of three independent experiments. **c** Neurovirulence of FSS13025 and MR766 in CD-1 mice. Neonatal mice were intracranially (i.c.) inoculated with either 1 PFU of viruses or PBS, and mortality was observed over a 3-week course. FSS13025: n=10; MR766: n=14; PBS: n=10. (n, number of mice). Log-rank test was performed for statistical analysis. ****, $P < 0.0001$. Source data are provided as a Source Data file.

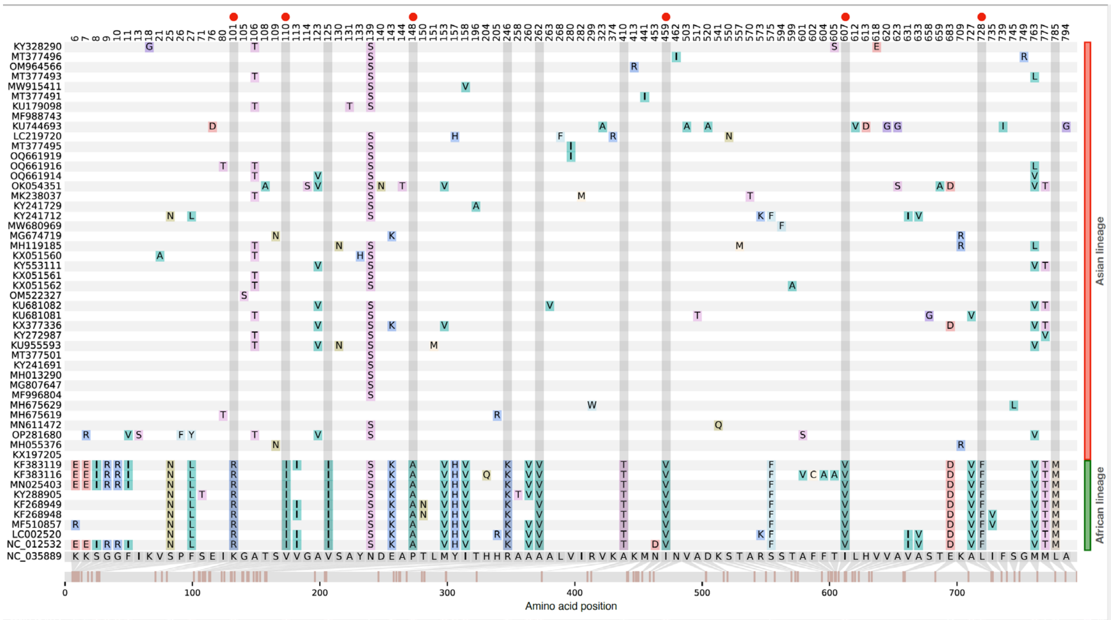

Fig. S2. **Comparative Analysis of Amino Acid Sequences in the preM/E Region of African and Asian-Lineage Zika Virus Strains.** The collection of 52 Zika viral strains, consisting of 43 Asian lineage and 9 African lineage representatives, was used to serve as the representative sequence for analysis. The NCBI accession ID was list in the left side of the plot and in the bottom is a bar that indicate the amino acid position. The NCBI reference sequence NC_035889 was used as the reference sequence for other 51 sequences. Sites with amino acid type different from the reference sequence was labeled as brown in the amino acid bar and the detailed amino acid in this site was shown in the row of each viral strain. For all these sites that have varied amino acid with reference sequence, a total of 11 sites exhibited a high degree of conservatism between Asian lineage and African lineage. All the African lineage viruses have one type of amino acid while the Asian lineage viruses have another. These sites were heighted with a gray background. The 6 sites that were selected for further analysis were labeled with a red node, located at the top of the corresponding site number.
