# Supplementary material for: A single amino acid substitution in the capsid protein of Zika virus contributes to a neurovirulent phenotype
Source: Nat Commun. 2023 Oct 26;14:6832. doi: 10.1038/s41467-023-42676-7 (PMC10603150; doi:10.1038/s41467-023-42676-7)
Supplement: Supplementary file 3 — Reporting Summary [file 41467_2023_42676_MOESM3_ESM.pdf]

nature portfolio

Corresponding author(s): Cheng-Feng Qin

Last updated by author(s): Sep 17, 2023

Reporting Summary

Nature Portfolio wishes to improve the reproducibility of the work that we publish. This form provides structure for consistency and transparency in reporting. For further information on Nature Portfolio policies, see our Editorial Policies and the Editorial Policy Checklist.

Statistics

For all statistical analyses, confirm that the following items are present in the figure legend, table legend, main text, or Methods section.

n/a	Confirmed	
☐	☒	The exact sample size (*n*) for each experimental group/condition, given as a discrete number and unit of measurement
☐	☒	A statement on whether measurements were taken from distinct samples or whether the same sample was measured repeatedly
☐	☒	The statistical test(s) used AND whether they are one- or two-sided<br>*Only common tests should be described solely by name; describe more complex techniques in the Methods section.*
☒	☐	A description of all covariates tested
☒	☐	A description of any assumptions or corrections, such as tests of normality and adjustment for multiple comparisons
☐	☒	A full description of the statistical parameters including central tendency (e.g. means) or other basic estimates (e.g. regression coefficient) AND variation (e.g. standard deviation) or associated estimates of uncertainty (e.g. confidence intervals)
☐	☒	For null hypothesis testing, the test statistic (e.g. *F*, *t*, *r*) with confidence intervals, effect sizes, degrees of freedom and *P* value noted<br>*Give P values as exact values whenever suitable.*
☒	☐	For Bayesian analysis, information on the choice of priors and Markov chain Monte Carlo settings
☒	☐	For hierarchical and complex designs, identification of the appropriate level for tests and full reporting of outcomes
☒	☐	Estimates of effect sizes (e.g. Cohen's *d*, Pearson's *r*), indicating how they were calculated

Our web collection on statistics for biologists contains articles on many of the points above.

Software and code

Policy information about availability of computer code

Data collection	Immunofluonrescence images were acquired using Olympus BX51 microscope equipped with a DP72 camera.<br>Origin iTC200 software (Malvern Instrument Ltd.)<br>CD-hit program (v4.8.1)<br>IQ-Tree (v1.6.12)<br>ImageJ software<br>Prism software (GraphPad)
Data analysis	The data from ITC assay were analyzed by using Origin iTC200 software (Malvern Instrument Ltd.). The CD-hit program (v4.8.1) was utilized, with a sequence identity threshold of 0.995, to select representative genomes of the Asian lineage. The maximum likelihood phylogenetic tree of ZIKV complete sequences was constructed using IQ-Tree (v1.6.12) with 1000 ultrafast bootstrap test. Quantification of bands of western blotting was performed using ImageJ software. The remaining data were analyzed using Prism software (GraphPad).

For manuscripts utilizing custom algorithms or software that are central to the research but not yet described in published literature, software must be made available to editors and reviewers. We strongly encourage code deposition in a community repository (e.g. GitHub). See the Nature Portfolio guidelines for submitting code & software for further information.

Data

Policy information about availability of data

All manuscripts must include a data availability statement. This statement should provide the following information, where applicable:

- Accession codes, unique identifiers, or web links for publicly available datasets
- A description of any restrictions on data availability
- For clinical datasets or third party data, please ensure that the statement adheres to our policy

Materials generated in this study will be made available on request and may require a material transfer agreement. Genomic sequence of the viruses used in this study are available in GenBank (https://www.ncbi.nlm.nih.gov/genbank) with corresponding accession codes.

Research involving human participants, their data, or biological material

Policy information about studies with human participants or human data. See also policy information about sex, gender (identity/presentation), and sexual orientation and race, ethnicity and racism.

Reporting on sex and gender	Not applicable.
Reporting on race, ethnicity, or other socially relevant groupings	Not applicable.
Population characteristics	Not applicable.
Recruitment	Not applicable.
Ethics oversight	Not applicable.

Note that full information on the approval of the study protocol must also be provided in the manuscript.

Field-specific reporting

Please select the one below that is the best fit for your research. If you are not sure, read the appropriate sections before making your selection.

- [x] Life sciences
- [ ] Behavioural & social sciences
- [ ] Ecological, evolutionary & environmental sciences

For a reference copy of the document with all sections, see nature.com/documents/nr-reporting-summary-flat.pdf

Life sciences study design

All studies must disclose on these points even when the disclosure is negative.

Sample size	All sample sizes were stated in the manuscript and figure legends. Sample size was chosen based on previous experience and standards in the field.
Data exclusions	No data was excluded from the analysis.
Replication	All cell culture experiments were repeated multiple independent times. In vivo experiments were performed with independent repeat experiments.
Randomization	For in vitro experiments of this study: randomization is not relevant for these in vitro studies. Positive and negative controls are always included in all experiments to ensure consistency among experiments. The CD-1 dams were randomly assigned to different groups, and the newborn litter of each dam was maintained in the same group (cage) with their mother.
Blinding	Investigators were not blinded during data collection or analysis.

Reporting for specific materials, systems and methods

We require information from authors about some types of materials, experimental systems and methods used in many studies. Here, indicate whether each material, system or method listed is relevant to your study. If you are not sure if a list item applies to your research, read the appropriate section before selecting a response.

Materials & experimental systems

n/a	Involved in the study
☐	☒ Antibodies
☐	☒ Eukaryotic cell lines
☒	☐ Palaeontology and archaeology
☐	☒ Animals and other organisms
☒	☐ Clinical data
☒	☐ Dual use research of concern
☒	☐ Plants

Methods

n/a	Involved in the study
☒	☐ ChIP-seq
☒	☐ Flow cytometry
☒	☐ MRI-based neuroimaging

Antibodies

Antibodies used	Anti-Zika ENV mAb (BF-1176-56, BioFront Technologies); Anti-GAPDH antibodies (MA5-15738, Invitrogen,); anti-C antibodies (GTX133317, GeneTex).
Validation	All antibodies used in this study were commercially available. They were validated by the manufacturer for use in immunofluorescent assay and we determined the optimal concentration for our assays.

Eukaryotic cell lines

Policy information about cell lines and Sex and Gender in Research

Cell line source(s)	Cell lines used in this study were obtained from ATCC (BHK-21 (CCL-10) ,Vero (CCL-81), C6/36 (CRL-1660), and Aag2 cells (ATCC, CCL-125)), or kindly provided by S. Bao (Cleveland Clinic) (hNPC cells).
Authentication	None of the cell lines used were authenticated.
Mycoplasma contamination	All cells were tested and confirmed mycoplasma-negative.
Commonly misidentified lines (See ICLAC register)	No commonly misidentified cell lines were used in the study.

Animals and other research organisms

Policy information about studies involving animals; ARRIVE guidelines recommended for reporting animal research, and Sex and Gender in Research

Laboratory animals	CD-1 neonatal mice were used in this study.
Wild animals	No wild animals were involved in this study.
Reporting on sex	Not applicable.
Field-collected samples	The study did not involve sample collection from the field.
Ethics oversight	All animal experiments were performed in strict accordance with the guidelines of the Chinese Regulations of Laboratory Animals (Ministry of Science and Technology of People's Republic of China) and Laboratory Animal-Requirements of Environment and Housing Facilities (GB 14925-2010, National Laboratory Animal Standardization Technical Committee). All procedures were approved by the Animal Experiment Committee of Laboratory Animal Center, AMMS, China (IACUC-IME-2021-010).

Note that full information on the approval of the study protocol must also be provided in the manuscript.
